# Internalizing Mental Health Disorders and Emotion Regulation: A Comparative and Mediational Study of Older Adults With and Without a History of Complex Trauma Exposure

**DOI:** 10.3389/fpsyg.2022.820345

**Published:** 2022-06-23

**Authors:** Viviane Pfluger, Shauna L. Rohner, Carla M. Eising, Andreas Maercker, Myriam V. Thoma

**Affiliations:** ^1^Psychopathology and Clinical Intervention, Institute of Psychology, University of Zurich, Zurich, Switzerland; ^2^University Research Priority Program Dynamics of Healthy Ageing, University of Zurich, Zurich, Switzerland

**Keywords:** anxiety, complex trauma exposure, childhood, depression, emotion regulation, mental health, older adults, adolescence

## Abstract

Individuals with complex trauma exposure (CTE) in early life (i.e., childhood/adolescence) are at heightened risk for developing problems in various domains of functioning. As such, CTE has repeatedly been linked to internalizing mental health disorders, such as depression and anxiety, as well as emotion dysregulation across the lifespan. While these correlates of CTE are comparatively well studied up to middle adulthood, they are insufficiently studied in older adulthood. Therefore, this study aimed to (a) compare Swiss older adults with and without a CTE history regarding current and lifetime internalizing mental health disorders and emotion regulation strategies; and (b) to examine the potential mediating role of emotion regulation in the mental health disparities between these groups. A total of *N* = 257 participants (age = 49–95 years; 46.3% female) were assessed in a retrospective, cross-sectional study, using two face-to-face interviews. The CTE group (*n* = 161; *M*_age_ = 69.66 years, 48.4% female) presented with significantly more current and lifetime internalizing mental health disorders than the non-affected (nCTE) group (*n* = 96; *M*_age_ = 72.49 years, 42.7% female). The CTE group showed significantly higher emotion suppression and lower emotion reappraisal compared to the nCTE group. Mediation analysis revealed that the two emotion regulation strategies were significant mediators between CTE history and internalizing mental health disorders. Findings emphasize the relevance of emotion (dys-)regulation in understanding mental health disparities in older age and deciding about treatment strategies. Research and practice should pay more attention to the needs of this high-risk group of older individuals.

## Introduction

Potentially traumatic or adverse events in childhood and adolescence, such as child maltreatment, are common worldwide. Global self-report estimates suggest that up to 363 out of every 1,000 individuals may have experienced at least one type of maltreatment during childhood or adolescence (Stoltenborgh et al., 2015). Such child maltreatment often occurs within the caregiving system (i.e., both, familial and formal caregiving systems; e.g., Pinheiro, 2006). In addition, many affected minors do not experience only a single type of maltreatment (e.g., physical abuse, emotional neglect), but are exposed to multiple types of abuse and neglect (Scher et al., 2004; Green et al., 2010). This accumulation of maltreatment experiences within the caregiving system has been referred to as complex trauma exposure (CTE; Kisiel et al., 2009; Greeson et al., 2011), and will henceforth be used to describe exposure to at least two types of maltreatment in childhood and/or adolescence.

Exposure to complex trauma in childhood and/or adolescence has been associated with a range of negative health correlates across the life-course, including the development of mental health disorders (e.g., Cook et al., 2005; Spinazzola et al., 2005; Kisiel et al., 2009). Furthermore, ample evidence indicates that CTE in childhood and/or adolescence is associated with (the onset of) internalizing mental health disorders, such as depressive and anxiety disorders, across the lifespan. For instance, in the *short-term* (i.e., up to adolescence), children and adolescents with a CTE history have been shown to present with significantly high(er) rates of internalizing mental health problems, including depressive and anxiety symptoms, major depression, separation anxiety disorder, generalized anxiety disorder, panic disorder, and phobic disorders (e.g., Ford et al., 2010; Choi and Oh, 2014; Kroska et al., 2018; Haahr-Pedersen et al., 2020; Lewis et al., 2021). Additionally, in a comparative study of adolescents living in foster care, a history of CTE was found to increase the odds of having internalizing mental health problems in youth by 60% compared to those with other traumatic backgrounds (Greeson et al., 2011).

Similarly, existing studies on the *mid-term* (i.e., up to middle-age) mental health sequelae of CTE also indicate a high mental health burden due to clinically relevant internalizing mental health problems. Adults formerly affected by CTE have been shown to present with significantly high(er) rates of depressive and anxiety symptoms, as well as major depression, minor depression, dysthymia, generalized anxiety disorder, panic disorder, and phobic disorders (e.g., Chapman et al., 2004; Green et al., 2010; Putnam et al., 2013; Huh et al., 2017; Giraldo Gallo et al., 2018). For instance, a meta-analysis of 184 studies showed that adults who experienced one type of child maltreatment were 2.81 times more likely to develop a depressive disorder in adulthood; whereas for those with a CTE history, the odds increased to 3.61 (Nelson et al., 2017). In addition, a prospective, population-based cohort study investigated mental health disorders in adults with cumulative and single childhood trauma experiences. Between all of the investigated mental health disorders, odds for internalizing mental health disorders were among the highest for adults with a cumulative childhood trauma history: 1.7 for depressive disorders and 1.4 for anxiety disorders compared to 1.5 for any mental health disorder (Copeland et al., 2018). Moreover, a longitudinal 25-year study on the association between child maltreatment and internalizing mental health disorders across adulthood revealed a significantly elevated risk for individuals with a CTE history, compared to those with no or low child maltreatment history (Rapsey et al., 2019). This finding provides evidence for a long-lasting vulnerability and emphasizes the need to also consider CTE mental health sequelae from a *long-term* (i.e., in older adulthood) and a *lifespan* (i.e., lifetime disorders) perspective.

However, research on the long-term mental health correlates of CTE in older adults is scarce and there is a lack of studies focusing on internalizing mental health disorders in older adults with and without a history of CTE. Nonetheless, some studies have investigated the association between child maltreatment and mental ill-health in older samples. For instance, a review by Maschi et al. (2013) found that depressive and anxiety symptoms and disorders in older age are among the most often documented long-term correlates of child maltreatment. However, the studies in this review did not explicitly examine cumulative child maltreatment. Nevertheless, based on such related research, it could be assumed that CTE may also be linked to an elevated risk for internalizing mental health disorders in older adulthood. However, further research is needed to investigate this in older adult samples. In addition, research is also needed on the potential underlying processes involved in the development of internalizing mental health disorder in CTE survivors.

One potential process underpinning the lasting mental health effects of CTE may be the dysregulation of emotions. Emotion regulation refers to the ability to recognize, monitor, express, and modify emotional reactions in a way that facilitates adaptive functioning (Gratz and Roemer, 2004). Applying a developmental perspective, several studies indicate that emotion regulation strategies develop in the early stages of life and primarily within the context of an emotional relationship, such as the caregiving context (e.g., *via* observation; parenting practice, such as the validation of emotions; or the emotional atmosphere at home; Morris et al., 2007; Ehring and Quack, 2010). However, in a family environment of child maltreatment, children are exposed to caregivers who cannot satisfy this educational task appropriately. Child maltreatment, and CTE in particular, may therefore lead to emotion regulation difficulties by hampering the development of adaptive strategies (e.g., distraction, reappraisal, acceptance), while fostering the development of maladaptive strategies (e.g., self-devaluation, suppression, withdrawal; Cook et al., 2005; Spinazzola et al., 2005; D'Andrea et al., 2012). In support of this, research has repeatedly documented medium to high correlations between cumulative child maltreatment and emotion regulation difficulties in samples of diverse ages (for children/adolescents examples, see Dunn et al., 2018; Hébert et al., 2018; Haselgruber et al., 2021; for adults examples, see Carvalho Fernando et al., 2014; Jennissen et al., 2016; Dutcher et al., 2017). In addition, recent evidence comes from a comparative study on emotion regulation in adolescents and young adults (aged 12–22 years). Results showed that emotion regulation difficulties in CTE survivors significantly differed (i.e., more emotion regulation difficulties) from no or low maltreatment survivors (Henschel et al., 2019). However, as no existing studies focus solely on an older sample, it remains unclear whether differences in emotion regulation difficulties between individuals with and without a CTE history are also evident into older adulthood. This is particularly relevant, as previous research has not only emphasized the high relevance of emotion regulation in older age (Carstensen et al., 1999), but has also provided empirical evidence on changes in the use of emotion regulation strategies across adulthood and into older age (e.g., Urry and Gross, 2010; Eldesouky and English, 2018), such as an age-related increase in emotion reappraisal (e.g., Masumoto et al., 2016).

With regard to the potential underlying mechanism, emotion regulation has repeatedly been found to mediate the relationship between child maltreatment and subsequent psychopathology (e.g., Aldao et al., 2010; Kim and Cicchetti, 2010; Knefel et al., 2019; Weissman et al., 2019); including various internalizing mental health problems (e.g., Choi and Oh, 2014; Heleniak et al., 2016; Cloitre et al., 2019). For instance, a clinical study assessed adults (aged 18–65 years) diagnosed with at least one internalizing mental health disorder (Huh et al., 2017). Results found that maladaptive emotion regulation mediated the relationship between child maltreatment and depressive and anxiety symptomatology (investigated as two distinct variables). Furthermore, in one of the few to include both adaptive and maladaptive emotion regulation, Haselgruber et al. (2020) showed both emotion regulation strategies to mediate the relationship between cumulative child maltreatment and internalizing mental health symptoms. While the existing studies provide valuable insight into this mediating role of emotion regulation, some aspects of this interplay have been overlooked. For instance, existing studies have mainly used a specific type of child maltreatment (i.e., childhood sexual abuse; for example, see Choi and Oh, 2014) or have included child maltreatment as a continuous variable in the mediation model (i.e., mediation analysis focusing on whether a mediator variable may explain a continuous relationship between two variables, considering variables as a whole; for examples, see Hopfinger et al., 2016; Huh et al., 2017). As such analyses do not aim to address the comparison of two specific groups which are differentially affected by child maltreatment, previous studies do not provide sufficient empirical evidence that differences in emotion regulation can explain disparities in internalizing mental health problems between individuals with and without a CTE history (i.e., CTE used as a dichotomous variable). Furthermore, existing mediation studies have mainly focused on a single mental health disorder category (i.e., either depressive or anxiety symptoms/disorders), assessed within a particular life stage, such as childhood (Hébert et al., 2018). In doing so, this approach neglects a combined perspective of several developmental stages (i.e., lifetime disorders), as well as a group of mental health disorders (i.e., internalizing mental health disorders). These considerations are crucial, given the relatively high comorbidity of depressive and anxiety disorders across the lifespan (Kaufman and Charney, 2000; Cummings et al., 2014).

Taken together, research is lacking on the long-term correlates of CTE in childhood and/or adolescence for mental health and emotion regulation. Given the repeated evidence of these correlates in the short- and mid-term and the significant role of emotion regulation for wellbeing in older adults, as suggested by the socioemotional selectivity theory (Carstensen et al., 1999) and the wellbeing paradox (Hansen and Slagsvold, 2012), it is of utmost importance to (further) include the steadily growing population of older adults into this research. Yet, it is unclear to what extent the above-described age-related changes in emotion regulation relate to potential CTE-related changes in emotion regulation up to older adulthood and what this may imply regarding the development of internalizing mental health disorders in older age. Both, amplification, and mitigation processes are possible. Furthermore, by focusing on internalizing mental health disorders, valuable information can be obtained on a dominant mental health disorder category in older age (e.g., Mühlig et al., 2015) as well as a leading cause of the global burden of disease (Whiteford et al., 2013). Moreover, combining this research with a lifespan perspective could provide knowledge on the still insufficiently understood topic of mental health disparities in older age (e.g., Thoma et al., 2021). Finally, despite the broad consensus between researchers and practitioners for a dose-response relationship of child maltreatment and psychopathology (Cook et al., 2005; Putnam et al., 2013), comparative studies are scarce (e.g., Lewis et al., 2021; Pfluger et al., 2021). Further comparative evidence is needed, particularly on the long-term correlates of CTE, to coherently integrate findings into the existing trauma literature.

Therefore, the current study aimed to examine long-term sequelae of CTE in childhood and/or adolescence in comparison to a control group of older adults with only single or no trauma exposure in childhood and/or adolescence. Specifically, these two groups will be compared with regard to current and lifetime internalizing mental health disorders, as well as adaptive and maladaptive emotion regulation strategies. Regarding the latter, the focus relies on emotion reappraisal and emotion suppression, as two of the most often investigated emotion regulation strategies (Preece et al., 2021). It is expected that individuals with a CTE history in childhood and/or adolescence will report more current and lifetime internalizing mental health disorders and more difficulties with emotion regulation (i.e., more suppression and less reappraisal) compared to the control group. This study further aims to examine the potentially mediating role of emotion regulation in the relationship between CTE history and internalizing mental health disorders. It is expected that the emotion regulation strategies suppression (i.e., negative effect) and reappraisal (i.e., positive effect) will both significantly mediate the relationship between CTE history (no/yes) and internalizing mental health disorders later in life.

## Materials and Methods

This study was conducted as part of a larger project on differential aging trajectories in high-risk individuals with past experiences of early adversity (see, Thoma et al., 2021). The study protocol is in accordance with the Declaration of Helsinki and was approved by the Ethics Committee of the Faculty of Arts and Social Sciences at the University of Zurich (ID: 19.4.3).

### Recruitment

Adults aged 50 years (i.e., born in 1969) and older and with Swiss German as their native language were recruited between July and December 2019 via study flyers. Flyers were distributed at places specifically aimed at older citizens (e.g., senior leisure clubs), as well as at various public places (e.g., supermarkets, pharmacies) in the German-speaking region of Switzerland. In addition, flyers were also sent to individuals from a study pool of the affiliated University Research Priority Program, Dynamics of Healthy Ageing of the University of Zurich. Recruitment also aimed to include a particular sample of Swiss older adults, who were affected by compulsory social measures and/or placements (CSMP) during their childhood and/or adolescence. Research has indicated that survivors of such child welfare practices in the last century have an increased risk of exposure to (complex) traumatic experiences in childhood/adolescence (e.g., Ferguson, 2007; Thoma et al., 2021). These individuals were mainly recruited using a contact list provided by the Swiss Federal Office of Justice (2020). Additional recruitment methods for this high-risk group included word-of-mouth recommendations and contacting publicly active CSMP survivors.

### Procedure

Eligible participants took part in two assessments, each lasting a maximum of 120 min and conducted by trained interviewers at the university or, if preferred, at the participant's home. Written informed consent was obtained from all participants before starting the first assessment, which consisted of a structured clinical interview to assess current and lifetime mental health disorders. The second assessment was scheduled within 1 week of the first assessment and collected data on traumatic experiences in childhood and adolescence, lifetime traumata, current PTSD symptomatology, and various psychological resources. Between the two assessments, participants also completed questionnaires covering a range of topics, such as demographic information and emotion regulation. After completing the second assessment, all participants were reimbursed for their participation. For a more extensive description of the study procedure, see Thoma et al. (2021).

The mental health data of this study has been previously used in other publications. The publication by Thoma et al. (2021) investigated mental health disparities in a risk group of older CSMP survivors compared to a non-affected control group. The publication by Pfluger et al. (2021) investigated overall psychopathology and stress coping in older individuals with and without a history of CTE.

### Measures

A broad set of psychometric measures were assessed in the larger project. The following section presents those relevant for the current study regarding complex trauma, emotion regulation, internalizing mental health disorders, and covariates.

### Complex Trauma Exposure History

CTE in childhood and/or adolescence was assessed with the German version of the Childhood Trauma Questionnaire (CTQ; Bernstein and Fink, 1998; Gast et al., 2001). The CTQ is a self-report questionnaire that assesses types of abuse (emotional, physical, and sexual) and neglect (emotional and physical). The five subscales consist of five items each, rated on a 5-point Likert scale ranging from 1 (*not at all*) to 5 (*very often*). Subscale scores range from 5 to 25, with higher scores indicating more traumatic or adverse experiences in childhood and/or adolescence. The severity of each trauma/adversity type was calculated as proposed by Häuser et al. (2011), with each trauma/adversity type considered present if the level or higher was indicated. To build groups with and without a CTE history, CTE was operationalized as the presence of at least two interpersonal traumatic/adverse experiences in childhood and/or adolescence at an actionable level (i.e., severity ratings moderate to extreme; for example, see Kisiel et al., 2009). In the present study, all five subscales showed high internal consistency (emotional abuse: α = 0.83; physical abuse: α = 0.83; sexual abuse: α = 0.96; emotional neglect: α = 0.88; physical neglect: α = 0.78).

### Emotion Regulation Strategies

Emotion regulation strategies were assessed with the German version of the Emotion Regulation Questionnaire (ERQ; Gross and John, 2003). The ERQ is a self-report questionnaire assessing the emotion regulation strategies suppression (e.g., “*I keep emotions to myself* ”) and reappraisal (e.g., “*I control my emotions by changing the way I think about the situation I am in*”), with four and six items, respectively. Items are rated on a 7-point Likert scale ranging from 1 (*strongly disagree*) to 7 (*strongly agree*). Subscale scores range from 4 to 28 for emotion suppression and from 6 to 42 for emotion reappraisal. In the present study, both subscales showed high internal consistency (suppression: α = 0.75; reappraisal: α = 0.81).

### Internalizing Mental Health Disorders

To assess internalizing mental health disorders, the German structured clinical interview for diagnosing mental health disorders was used (DIPS; Margraf et al., 2017a,b). The DIPS allows for the diagnosis of current and lifetime (i.e., at any time in the past adult life) mental health disorders according to the DSM-5. The individual level of current and lifetime internalizing mental health disorders was computed, as well as an index score (i.e., total number of current and lifetime mental health diagnoses). By including both, current and lifetime mental health disorders, the study aimed to present a full(er) picture of the mental health burden across the adult lifespan of older individuals. The following internalizing mental health disorders were assessed: Anxiety disorders (separation anxiety, panic disorder, agoraphobia, social phobia, specific phobia, generalized anxiety disorder) and depressive disorders (dysthymia, major depression). The index score ranges from 0 to 16, with higher scores indicating more mental health disorders.

### Covariates

#### Lifetime Trauma

Lifetime trauma was assessed using a list of 18 potentially traumatic experiences across the lifespan included in the PTSD section of the DIPS (Margraf et al., 2017a,b). For each of the 18 traumatic experiences (e.g., sexual violence in adulthood, serious accident), participants indicated whether they had experienced it (yes = 1) or not (no = 0). The score ranges from 0 to 18, with higher scores indicating more lifetime trauma.

#### PTSD Symptomatology

Current PTSD symptomatology was assessed using the German version of the International Trauma Questionnaire (ITQ; Cloitre et al., 2018). The ITQ is a self-report questionnaire that assess ICD-11 PTSD symptomatology, with six items rated on a 5-point Likert scale ranging from 0 (*not at all*) to 4 (*extremely*). Scores range from 0 to 24. The present study showed high internal consistency (α = 0.87).

### Data Analysis

All statistical analyses were conducted with IBM Statistical Package for Social Sciences (SPSS) version 26 (IBM Corp, 2020). Pre-processing of the data involved missing values analyses, descriptive analyses, and bivariate correlation analysis between all variables of interest. An expectation maximization imputation algorithm with 25 iterations was used to replace missing values (<1% for each questionnaire).

One-way analyses of covariance (ANCOVA) were then conducted to compare levels of internalizing mental health disorders and emotion regulation strategies between groups with and without a CTE history. Taking into account the repellent literature on gender differences in internalizing mental health disorders (e.g., Boyd et al., 2015; Salk et al., 2017), gender was included to the analysis, but only as a side aspect. To exploratively investigate a potential gender effect, the above-mentioned analysis on internalizing mental health disorders was re-run as a two-way ANCOVA with CTE (no/yes) and gender (male/female) as independent variables and CTE^*^ gender as an interaction term. In addition, group differences in the included mental health disorders (both current and lifetime) were tested using Pearson's Chi-squared test.

Finally, a mediation analysis was run to investigate if the effect of CTE on internalizing mental health disorders was mediated by emotion suppression and emotion reappraisal. Both potential mediators were included in the same model, as they were only slightly correlated (*r* = 0.058). Parallel mediation analysis (model 4) was performed using the PROCESS version 3.0 macro for SPSS (Hayes, 2013), which uses ordinary least squares regression, yielding unstandardized path coefficients for total, direct, and indirect effects. Confidence intervals and standard errors for all parameter estimates were produced using 5,000 bootstrapped samples. Effects were deemed significant when the confidence interval did not include zero.

Age, the number of lifetime trauma, and current PTSD symptomatology were included as covariates throughout the analyses on emotion regulation. Including the number of lifetime trauma and PTSD symptomatology allowed for the controlling of the potential confounding effect that emotion regulation would be higher due to an elevated number of lifetime trauma and PTSD symptomatology (for example, see Ehring and Quack, 2010; Shepherd and Wild, 2014). Including age allowed for the controlling of the potential confounding effect that group differences in emotion regulation would be biased by an age-related adaptation of emotion regulation strategies sometimes observed in older adults (for example, see Eldesouky and English, 2018). In addition, age and the number of lifetime trauma were also included as covariates in the analyses on internalizing mental health disorders as well as on the potential mediating role of emotion regulation. This allowed for the controlling of the potential confounding effect that differences in mental health would be higher due to higher age and an elevated number of lifetime trauma (e.g., Dulin and Passmore, 2010).

## Results

### Sample Characteristics

The following sample characteristics were assessed: demographics and trauma history.

### Demographical Analysis

The study sample consisted of *N* = 257 participants aged 49–95 years (*M*_age_ = 70.72 years, *SD* = 11.08, 46.3% female). A total of 161 participants (62.7%) met the criteria for CTE and 96 participants (37.3%) did not meet these criteria (the nCTE group). The two groups were comparable with regard to the demographics of sex, relationship status, and employment status; but differed significantly (*p* < 0.05) with respect to age, education, and income (see Table 1 for sample characteristics).

**Table 1:** Sample characteristics.

	**Total sample**	**CT**	**nCT**	**Group comparison**
	**(*N* = 257)**	**(*n* = 161)**	**(*n* = 96)**	
Age (*M, SD*)^a^	70.72 (11.08)	69.66 (11.34)	72.49 (10.46)	*t*(255) = 1.992, *p* = 0.047
Sex (female) (%)	46.3	48.4	42.7	X^2^ = 0.797*, p* = 0.372
Relationship status (%)				
Single	12.5	13.0	11.5	X^2^ = 10.661*, p* = 0.059
In a relationship	11.3	13.0	8.3	
Married	41.2	36.6	49.0	
Separated	1.9	3.1	0	
Divorced	20.2	23.6	14.6	
Widowed	12.8	10.6	16.7	
Employment status (%)				
Employed	21.4	24.8	15.6	X^2^ = 5.044*, p* = 0.283
Unemployed	2.7	3.1	2.1	
Retired/pension	58.0	53.4	65.6	
Voluntary work	11.7	10.6	13.5	
Highest level of education (%)				
No education	2.3	3.1	1.0	X^2^ = 19.849*, p* = 0.006
Primary school	3.9	5.0	2.1	
Upper secondary school	10.5	13.7	5.2	
Secondary/High school	2.3	1.9	3.1	
Vocational job training	39.3	42.9	33.3	
Higher professional training	14.8	14.9	14.6	
University level	21.8	14.3	34.4	
Income class (per month) (%)				
<2001 Swiss Francs	15.2	20.5	6.3	X^2^ = 26.612*, p* < 0.001
2001–3330 Swiss Francs	19.8	25.5	10.4	
3331–4670 Swiss Francs	16.7	16.8	16.7	
>4670 Swiss Francs	46.7	35.4	65.6	

a *Range = 49–95 years*.

*CT, complex trauma group; nCT, no complex trauma group; M, mean; SD, standard deviation; X^2^, Pearson's Chi-squared test; t, two-sided t-test comparing complex trauma group with no complex trauma group; p, p-value*.

### Trauma History Analysis

On average, individuals in the CTE group reported *M* = 3.55 (*SD* = 1.03, range = 2–5) potentially traumatic experiences in childhood and/or adolescence. Within the CTE group, emotional neglect (93.8%) was the most prevalent trauma type, followed by physical neglect (85.7%), emotional abuse (59.0%), sexual abuse (58.6%), and physical abuse (58.4%). Table 2 displays the combined number of childhood and/or adolescence trauma, the number of lifetime trauma, as well as current PTSD symptomatology, separately for the CTE and nCTE groups. Groups significantly differed regarding the number of lifetime trauma (*t*(255) = −3.911, *p* < 0.001), and current PTSD symptoms (*t*(253) = −5.076, *p* < 0.001).

**Table 2:** Trauma history analysis and PTSD symptomatology.

	**Range**	**CTE** **(*n* = 161)**	**nCTE** **(*n* = 96)**
Overall number of childhood/ adolescence trauma *M* (*SD*)^a^	0–5	3.55 (1.03)	0.41 (0.49)
Number of types of childhood/adolescence trauma *n* (%)^a^			
No traumatic experience			57 (59.4)
One type of trauma			39 (40.6)
Two types of traumata		28 (17.4)	
Three types of traumata		53 (32.9)	
Four types of traumata		43 (26.7)	
Five types of traumata		37 (23.0)	
Number of lifetime trauma *M* (*SD*)^b^	0–18	5.94 (3.15)	4.46 (2.53)
PTSD symptomatology *M* (*SD*)^b^	0–24	6.27 (6.17)	2.57 (4.57)

a *Assessed with the CTQ, Childhood Trauma Questionnaire*.

b *Assessed with the ITQ, International Trauma Questionnaire; CTE, Complex trauma exposure group; nCTE, No complex trauma exposure group*.

### Group Comparisons of Internalizing Mental Health Disorders

Tables 3, 4 display detailed information on current and lifetime internalizing mental health disorders for the total sample, as well as separately for both groups. Across both groups, 29.7% of older adults presented with a current internalizing mental health disorder (CTE group: 36.9%; nCTE group: 17.7%); whereas 52.7% of older adults presented with a lifetime internalizing mental health disorder (CTE group: 60.0%; nCTE group: 40.6%). The most common *lifetime* mental health disorders in both groups were depressive disorders (i.e., major depression and dysthymia). The most common *current* mental health disorders in both groups were anxiety disorders (i.e., specific phobia and separation anxiety). Overall, 43.0% of the total sample had never experienced any of the assessed internalizing mental health disorders. Across both groups, females presented with higher (although not significant) prevalence rates for current and lifetime internalizing mental health disorders compared to males (females: 34.7% current, 61.0% lifetime; males: 25.4 % current, 45.7 % lifetime). For detailed prevalence rates for the CTE and nCTE groups, and separately for male and female participants, see Tables 3, 4.

**Table 3:** Current internalizing mental health disorders (diagnosis) and group comparisons.

**Current diagnosis, *n* (%)**	**CTE (*****n*** **= 161)**			**nCTE (*****n*** **= 96)**			**Group comparison** ^**a**^	
	**Total**	**Female**	**Male**	**Total**	**Female**	**Male**	**X^**2**^**	***p***
**Anxiety disorders**								
Separation anxiety	21 (13.0)	12 (15.4)	9 (10.8)	4 (4.2)	2 (4.9)	2 (3.6)	7.129	**
Panic disorder	5 (3.1)	3 (3.8)	2 (2.4)	3 (3.1)	1 (2.4)	2 (3.6)	0.001	1^b^
Agoraphobia	14 (8.7)	10 (12.8)	4 (4.8)	1 (1.0)	1 (2.4)		6.508	*
Social phobia	19 (11.8)	12 (15.4)	7 (8.4)	3 (3.1)	1 (2.4)	2 (3.6)	5.904	*
Specific phobia	22 (13.7)	14 (17.9)	8 (9.6)	7 (7.3)	5 (12.2)	2 (3.6)	4.697	*
Generalized anxiety	18 (11.2)	7 (9.0)	11 (13.3)	4 (4.2)	3 (7.3)	4 (7.3)	3.877	*
**Depressive disorders**									
Dysthymia	16 (9.9)	9 (11.5)	7 (8.4)	2 (2.1)	1 (2.4)	2 (3.6)	5.801	*
Major depression	14 (8.7)	8 (10.3)	6 (7.2)	1 (1.0)		1 (1.8)	6.508	*

a *Group comparison between the total number of each group*.

b *Computed using Fisher's exact test*.

CTE, complex trauma exposure group; nCTE, no complex trauma exposure group; X^2^= Pearson's Chi-squared test; p, p-value; *p < 0.05; **p < 0.01; ***p ≤ 0.001.

**Table 4:** Lifetime internalizing mental health disorders (diagnosis) and group comparisons.

**Lifetime diagnosis, *n* (%)**	**CTE (*****n*** **= 161)**			**nCTE (*****n*** **= 96)**			**Group comparison** ^**a**^	
	**Total**	**Female**	**Male**	**Total**	**Female**	**Male**	**X^**2**^**	***p***
**Anxiety disorders**								
Separation anxiety	24 (14.9)	14 (17.9)	10 (12.0)	6 (6.3)	1 (2.4)	5 (9.1)	4.501	*
Panic disorder	16 (9.9)	7 (9.0)	9 (10.8)	8 (8.3)	3 (7.3)	5 (9.1)	0.206	0.650
Agoraphobia	13 (8.1)	8 (10.3)	5 (6.0)	3 (3.1)	2 (4.9)	1 (1.8)	2.589	0.108
Social phobia	25 (15.5)	13 (16.7)	12 (14.5)	7 (7.3)	2 (4.9)	5 (9.1)	3.868	*
Specific phobia	20 (12.4)	12 (15.4)	8 (9.6)	4 (4.2)	3 (7.3)	1 (1.8)	4.958	*
Generalized anxiety	17 (10.6)	9 (11.5)	8 (9.6)	7 (7.3)	3 (7.3)	4 (7.3)	0.805	0.370
**Depressive disorders**								
Dysthymia	35 (21.7)	17 (21.8)	18 (21.7)	9 (9.4)	5 (12.2)	4 (7.3)	6.690	**
Major depression	65 (40.4)	35 (44.9)	30 (36.1)	25 (26.0)	14 (34.1)	11 (20.0)	5.782	*

a *Group comparison between the total number of each group*.

CTE, complex trauma exposure group; nCTE, no complex trauma exposure group; X^2^, Pearson's Chi-squared test; p, p-value; *p < 0.05; **p < 0.01; ***p ≤ 0.001.

Regarding the overall level of internalizing mental health disorders (i.e., the index score), the one-way ANCOVA showed a significantly higher mean score of internalizing mental health disorders in the CTE group (*M* = 2.15, *SD* = 2.62) compared to the nCTE group (*M* = 0.98, *SD* = 1.41; *F*(1,252) = 8.273, *p* = 0.004, $\eta_{\text{p}}^{2}$ = 0.032). In addition, both of the included covariates showed a significant positive effect on internalizing mental health disorders (number of lifetime trauma: *F*(1,252) = 6.747, *p* = 0.010, $\eta_{\text{p}}^{2}$ = 0.026; age: *F*(1,252) = 48.680, *p* < 0.001, $\eta_{\text{p}}^{2}$ = 0.162). Together, these results suggest that having a history of CTE, being of higher age, and reporting a higher number of lifetime trauma was associated with higher levels of internalizing mental health disorders. The two-way ANCOVA did not show a significant interaction effect between CTE history and gender on internalizing mental health disorders (*F*(1,251) = 1.749, *p* = 0.187, $\eta_{\text{p}}^{2}$ = 0.007), suggesting that the association between CTE and internalizing mental health disorders did not differ between female and male participants.

### Group Comparisons of Emotion Regulation Strategies

Results of the one-way ANCOVA showed a significantly higher mean subscale score for emotion suppression in the CTE group (*M* = 17.07, *SD* = 5.26) compared to the nCTE group (*M* = 15.21, *SD* = 5.67; *F*(1, 250) = 7.739, *p* = 0.006, $\eta_{\text{p}}^{2}$ = 0.030). Results also showed a significantly lower mean subscale score for emotion reappraisal in the CTE group (*M* = 26.38, *SD* = 7.36) compared to the nCTE group (*M* = 29.19, *SD* = 6.92; *F*(1, 250) = 4.129, *p* = 0.043, $\eta_{\text{p}}^{2}$ = 0.016). From the included covariates, age and current PTSD symptomatology showed a significant effect on both dependent variables, while the number of lifetime trauma did not show a significant effect on suppression (*p* = 0.07) nor on reappraisal (*p* = 0.60).

### Association Between Emotion Regulation and Internalizing Mental Health Disorders

**Figure 1:**
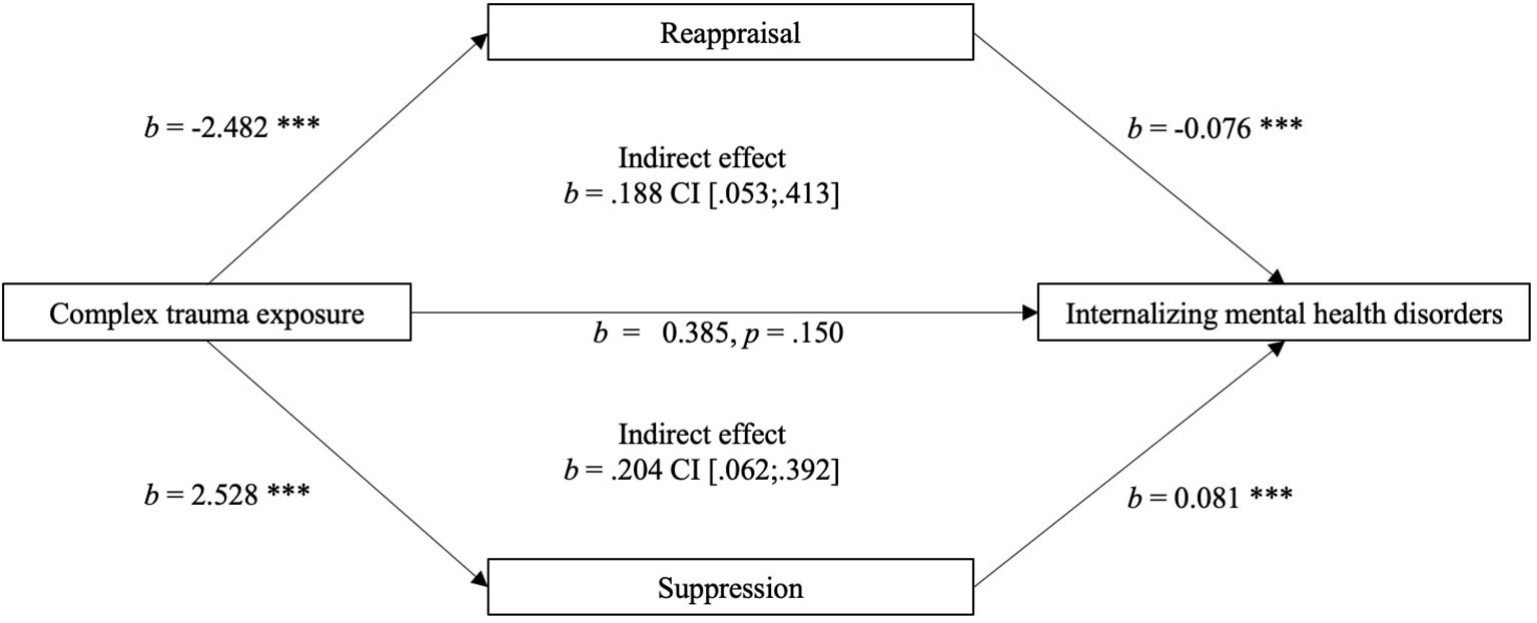
Parallel mediation model of the relationship between complex trauma exposure (Predictor) and internalizing mental health disorders (Outcome Variable), significantly mediated by emotion suppression and reappraisal (Mediators); with age and number of lifetime trauma as covariates.

The parallel mediation analysis showed that emotion suppression and emotion reappraisal fully mediated the relationship between CTE history and internalizing mental health disorders (current and lifetime). A significant total effect of CTE history on internalizing mental health disorders (i.e., the index score) was observed (*b* = 0.777, *t* = 2.876, *p* = 0.004), explaining 49.5% of the variance. When the two emotion regulation strategies (suppression and reappraisal) were included as (parallel) mediators in the model, a non-significant direct effect emerged (*b* = 0.385, *t* = 1.445, *p* = 0.150), explaining a greater percentage of the variance (57.2%). A significant indirect effect *via* both suppression (*b* = 0.204, 95% CI [0.060; 0.340]) and reappraisal (*b* = 0.188, 95% CI [0.035; 0.414]) was also observed, indicating that the two emotion regulation strategies significantly mediated the association between CTE history and internalizing mental health disorders. See Figure 1 for the full mediation model. The comparison of the standardized regression coefficients of both mediators showed a comparable effect on the association between CTE history and internalizing mental health disorders (suppression: β = 0.088; reappraisal: β = 0.081).

## Discussion

The present study examined the prevalence of current and lifetime internalizing mental health disorders and emotion (dys-)regulation in a sample of older adults with and without a history of CTE in childhood and/or adolescence. It further aimed to investigate the mediating role of emotion suppression and emotion reappraisal in the relationship between CTE in childhood and/or adolescence and internalizing mental health disorders later in life. Results showed that individuals with a history of CTE presented with a higher mental health burden regarding current and lifetime internalizing mental health disorders. Individuals affected by CTE also reported more disadvantageous emotion regulation than non-affected individuals, with higher levels of maladaptive emotion regulation and lower levels of adaptive emotion regulation. Both emotion regulation strategies significantly mediated the relationship between CTE history and internalizing mental health disorders, suggesting a potential underlying process for how early-life exposure to complex trauma may translate into mental ill-health over the life span.

### Internalizing Mental Health Disorders in Individuals With and Without a Complex Trauma Exposure History

In the present study, individuals with a history of CTE in childhood and/or adolescence showed a high(er) mental health burden, with meaningfully higher prevalence rates in various current and lifetime internalizing mental health disorders, compared to individuals with no CTE history. These included separation anxiety, social phobia, specific phobia, dysthymia, and major depression, as well as a significantly higher index score (i.e., total number of internalizing mental health disorders). The high(er) rates of current and lifetime internalizing mental health disorders in the CTE group were expected and corroborate existing literature on comparable, but younger, samples (e.g., Greeson et al., 2011; Nelson et al., 2017; Copeland et al., 2018). Moreover, the high prevalence rates within the CTE group emphasize the importance of not only considering trauma-related disorders in the context of (complex) trauma exposure (e.g., PTSD), but also specifically considering internalizing mental health disorders (e.g., Ford et al., 2010; Kroska et al., 2018; Humphreys et al., 2020). Given the comparatively high prevalence of *current* internalizing mental health disorders in this CTE group of older adults (i.e., 36.9%), this consideration is particularly relevant when investigating the relationship between (complex) trauma exposure and mental health from a lifespan perspective.

Regarding gender-related differences in mental health, comparisons revealed both within- and between-group differences. In the total sample, as well as in the two groups, females presented with more current and lifetime internalizing mental disorders than males. This finding is in line with existing literature on younger participants that shows a higher likelihood for females to develop internalizing mental health disorders and symptoms (e.g., Boyd et al., 2015; Salk et al., 2017). In the current study, gender differences in the prevalence rates of current and lifetime internalizing mental health disorders were smaller in the nCTE group than in the CTE group. This may suggest that particularly for females, a history of CTE can increase the probability of developing an internalizing mental health disorder. This is in line with a systematic review and meta-analysis by Giraldo Gallo et al. (2018), which found a tendency for a gender differences in the effect of childhood maltreatment on adulthood depression and anxiety. Future research should aim to build on these findings by using prospective longitudinal study designs to investigate incidence, recurrence, and comorbidity of internalizing mental health disorders in the aftermath of CTE.

### Emotion Regulation in Individuals With and Without a Complex Trauma Exposure History

The present study provides evidence for emotion dysregulation in older adult CTE survivors by identifying significant group differences with respect to emotion suppression and emotion reappraisal. Individuals with a history of CTE used significantly more suppression and significantly less reappraisal than individuals without such a history. These findings align with the few existing studies on emotion regulation in comparable, but younger, samples that depict emotion dysregulation as a part of the complex pattern of posttraumatic sequelae in CTE survivors (e.g., Hopfinger et al., 2016; Jennissen et al., 2016; Henschel et al., 2019). Thus, the present findings provide empirical evidence to suggest that difficulties in emotion regulation in CTE survivors may be identified up to old age (i.e., long-term sequelae). This finding is strengthened by two aspects: First, group differences were observed after controlling for potential confounding variables reported to impact emotion regulation, such as the number of lifetime trauma and current PTSD symptomatology (Ehring and Quack, 2010). Second, the mean subscale scores for emotion reappraisal and emotion suppression in the nCTE group was comparable with existing studies using the ERQ in older adult samples (e.g., Brady et al., 2019). This comparability suggests that the significant group differences observed in the current study were not due to a hyper-regulated control group. Taken together, there is evidence to assume that emotion dysregulation after CTE in childhood and/or adolescence can affect individuals of all ages, including older adulthood.

However, given the study's cross-sectional design, the data do not provide any information about emotion (dys-)regulation earlier in life. Given the research showing that emotion regulation patterns may change across adulthood (Eldesouky and English, 2018; Preece et al., 2021), particularly the age-related increase in emotion reappraisal (Masumoto et al., 2016); it could be that the group differences in the current study were more or less pronounced earlier in life than in older adulthood. To investigate this, longitudinal studies are needed to compare emotion regulation in individuals with and without a CTE history across several developmental stages. This would enhance understanding on the stability of emotion (dys-)regulation after CTE and potential variability over the life course. Beyond that, such longitudinal data could also provide further insight into the context-related effectiveness of emotion regulation strategies (i.e., each emotion regulation strategy can be either adaptive or maladaptive in different contexts, see Webb et al., 2012). Lastly, future research on the long-term emotional sequelae of CTE should also focus on potential alterations of emotion regulation flexibility, as this seems to be an integral component of healthy functioning and long-term adjustment (Bonanno et al., 2004; Eldesouky and English, 2018).

### The Mediating Role of Emotion Regulation in the Relationship Between Complex Trauma Exposure History and Mental Health

The investigated emotion regulation strategies, suppression and reappraisal, fully mediated the relationship between CTE history and current and lifetime internalizing mental health disorders (i.e., the index score). As the mediation analysis was run with the grouping variable as the independent variable, the results suggest that differences in suppression and reappraisal may help to explain the mental health disparities between individuals with and without a CTE history. This finding is in line with previous research in younger samples, emphasizing the relevance of emotion (dys-)regulation in the understanding of internalizing mental health problems in the aftermath of cumulative childhood trauma (e.g., Huh et al., 2017; Cloitre et al., 2019; Haselgruber et al., 2020). Furthermore, both mediators were comparably substantive and showed the expected associations: Suppression showed positive associations, and reappraisal showed negative associations with internalizing mental health disorders. However, as a composite index score of current and lifetime internalizing mental health disorders was applied in the current study, a potential obverse effect might have been disguised due to the elevated variable complexity. Therefore, future research is needed to evaluate whether a potential age-related alteration (i.e., a decrease) in the association of emotion suppression and reappraisal and mental health in individuals with a CTE history could exist or not.

### Strengths and Implications

The current study extends existing literature in several ways. First, the study adds to the limited body of research on the long-term correlates of CTE in childhood and/or adolescence and mental health in older adulthood (e.g., Pfluger et al., 2021). Specifically, this study builds on the literature by focusing on internalizing mental health disorders, a highly prevalent category of mental health disorders in the older adult population (Mühlig et al., 2015). Second, the study expands on the knowledge of emotion (dys-)regulation in the context of CTE history by adding a lifespan perspective. Extending this research into older adulthood is vital not only to enhance understanding of the potential (emotional) burden of individuals with a CTE history, but also the potential duration of vulnerability. Third, by comparing two groups of older adults, the study adds to the limited number of comparative studies on individuals with and without a history of CTE (e.g., Greeson et al., 2011; Lewis et al., 2021). As children and adolescents still face situations where CTE occurs (e.g., Stoltenborgh et al., 2015), this research is crucial in order to better understand the particular vulnerabilities and emotion regulation abilities of individuals with a CTE history compared to those with other or no traumatic experiences in childhood and/or adolescence. This knowledge would be highly relevant for preventive and therapeutic measures.

The study findings also have meaningful implications for clinical practice. For instance, the results support the notion of integrating detrimental childhood experiences into the understanding of the development of mental health disorders later in the life course. However, this link may not be obvious in every case and could become more difficult to distinguish with higher age (i.e., more distance from childhood/adolescence). Therefore, therapists can help patients to understand and navigate the connection between CTE experienced in childhood and/or adolescence and potential manifestations as depressive or anxiety disorders in older age. In addition, the study findings on the relevance of emotion regulation for internalizing mental health disorders also has connotations for clinical practice. For example, emotionally dysregulated trauma survivors could benefit from emotion-stabilization work before beginning trauma-focused treatment (e.g., Cloitre et al., 2012). This treatment approach could be applied to work with older patients with internalizing mental health disorders.

### Limitations

The current study also has some methodological limitations that must be noted. First, the retrospective assessment, particularly with distal experiences in an older sample, such as child maltreatment, may be affected by memory recall and retrieval bias (Sheikh, 2018). As group assignment was based on this retrospective data, this could also have led to an assignment bias. Furthermore, the cross-sectional study design prevents statements on causal inference. In addition, while a broad range of diagnoses were included in the larger project, the presence of comorbid diagnoses was not analyzed in the current study. This may be a relevant consideration, as recent research has shown that emotion regulation processes are involved in the co-occurrence of major depressive disorder and PTSD in individuals with traumatic experiences (Post et al., 2021). This cumulative perspective should be examined in future research. An additional limitation was that only two emotion regulation strategies were included in this study. Thus, only limited insight can be provided into the broad and multi-faceted construct of emotion regulation (e.g., Gratz and Roemer, 2004).

## Conclusion

Child maltreatment, and particularly CTE, is an extremely detrimental experience that has a high potential to affect mental health and self-regulation abilities across the life span (e.g., Cook et al., 2005; Pfluger et al., 2021). The present study investigated the long-term correlates of CTE in childhood and/or adolescence by focusing on internalizing mental health disorders and emotion regulation in two groups of Swiss older adults (i.e., a CTE group and a control group). Comparative analyses revealed a significantly higher current and lifetime mental health burden, as well as significantly more difficulties in regulating emotions, in survivors of CTE. Moreover, results suggest that differences in emotion regulation may provide a potential explanation for the mental health disparities found in the current study. Altogether, the study findings provide more elaborated insights into a highly vulnerable group of older adults that is often neglected in the research on complex Trauma exposure and mental health.

## Data Availability Statement

The raw data supporting the conclusions of this article will be made available by the authors, without undue reservation.

## Ethics Statement

The studies involving human participants were reviewed and approved by Ethics Committee of the Faculty of Arts and Social Sciences at the University of Zurich (ID:19.4.3). The patients/participants provided their written informed consent to participate in this study.

## Author Contributions

MT and SR conceived the idea for the study and were responsible for the conception and design of the study, managed the data collection. VP and CE were involved in data collection. Data analysis was performed by VP. The first draft of the manuscript was written by VP and all authors commented on previous versions of the manuscript. All authors read and approved the final manuscript.

## Funding

This publication is based on data from the study Differential Aging Trajectories in High-Risk Individuals with Past Experiences of Early Adversity, funded by the Swiss National Science Foundation, National Research Program 76, Welfare and Coercion—Past, Present and Future (grant no. 407640_177355/1). This study was also supported by the University Research Priority Program (URPP) Dynamics of Healthy Ageing at the University of Zurich, Switzerland.

## Conflict of Interest

The authors declare that the research was conducted in the absence of any commercial or financial relationships that could be construed as a potential conflict of interest.

## Publisher's Note

All claims expressed in this article are solely those of the authors and do not necessarily represent those of their affiliated organizations, or those of the publisher, the editors and the reviewers. Any product that may be evaluated in this article, or claim that may be made by its manufacturer, is not guaranteed or endorsed by the publisher.
